# Unusual Presentation of Primary Squamous Cell Carcinoma of Mandible

**DOI:** 10.1155/2016/9154309

**Published:** 2016-12-19

**Authors:** Karpagavalli Shanmugasundaram, Sathasiva Subramanian, Vaishnavi Vedam, Vimal Kumar

**Affiliations:** ^1^Department of Oral Medicine & Radiology, Saveetha Dental College, Saveetha University, Chennai, India; ^2^Department of Oral Medicine & Radiology, Faculty of Dental Sciences, Sri Ramachandra University, Chennai, India; ^3^Department of Oral Pathology, Faculty of Dentistry, Asian Institute of Medicine, Science & Technology (AIMST) University, Malaysia; ^4^Department of Oral Medicine & Radiology, Karpaga Vinayaga College of Dental Sciences, Chennai, India

## Abstract

Carcinoma arising primarily from the jaw is a locally aggressive lesion with poor prognosis. Primary intraosseous carcinoma (PIOC) lesion develops either de novo remnants of odontogenic epithelium, odontogenic cyst/tumor, epithelium remnants, or/and salivary gland residues. We describe very interesting case of primary intraosseous carcinoma of mandible. This extensive lesion was sent for oncological opinion and further management. Due to the uncertainty of diagnostic criteria of PIOC, only few cases of this lesion with a typical presentation have been reported. This article presents a case of primary intraosseous carcinoma with a unique appearance and detailed review stating its clinicopathological correlation.

## 1. Introduction

Primary intraosseous carcinoma (Pindborg; 1971) of the jaw is a rare malignant lesion ranging up to 6% of all malignant neoplasms of maxillofacial region [1, 2]. According to World Health Organization (WHO), International Histologic Classification of Tumors, Primary intraosseous carcinoma (PIOC) is a squamous cell carcinoma arising within the jaw that has no original connection with the surface epithelium of the oral mucosa, overlying skin, and antral or nasal mucosa developing from odontogenic epithelium or from a odontogenic cyst/tumor [3–5].

This article reports a case of aggressive primary intraosseous carcinoma invading the entire mandible with a unique presentation. The case findings have been thoroughly discussed correlating to the normal clinicopathological features of PIOC in oral and maxillofacial region.

## 2. Case Presentation

A sixty-six-year-old female visited the hospital with chief complaint of swelling in the lower left jaw region since 1 month. Swelling was insidious in onset and gradually progressed to the present size. The patient also gave history of reduced mouth opening since last 5 days. Past dental history revealed extraction of lower left back teeth in the same region 6 months ago with uneventful wound healing of the surgically explored site a month later.

On extra oral examination, facial asymmetry with diffuse irregular swelling was evident on the lower left side of the jaw measuring about 3 cm in size. On palpation swelling was warm, nontender, and firm in consistency. Restricted temporomandibular movements and paresthesia were evident (till the lower left chin area). Submandibular lymph nodes were hard and fixed to the underlying tissues.

**Figure 1:**
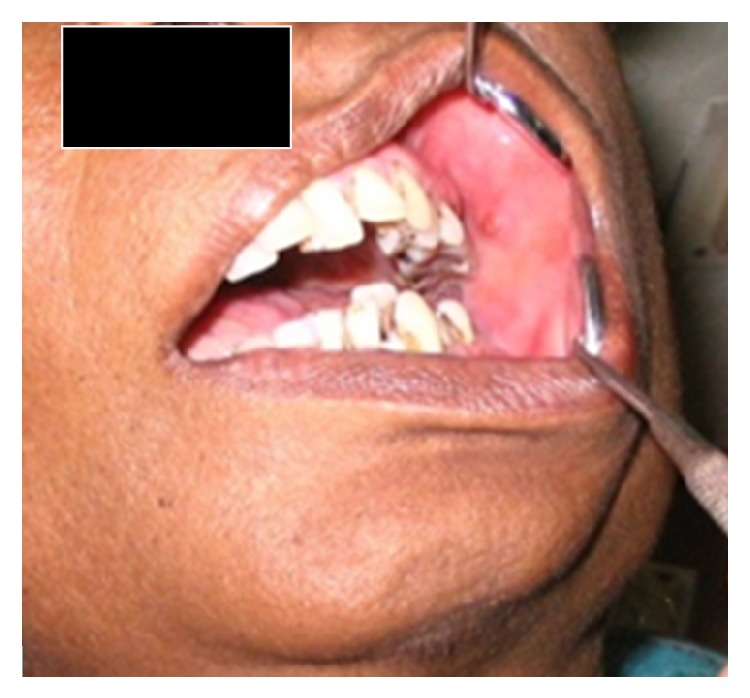
Intraoral view showing limited jaw movements with mild cortical plate expansion.

On intraoral examination, there was restricted mouth opening with a maximum interincisal distance between 11 and 41 measuring about 1 cm only. Mild tenderness with buccal cortical plate expansion in relation to 36,37 was seen. Provisional diagnosis of carcinoma of mandible and differential diagnosis of space infection, chronic osteomyelitis, and metastatic lesion were considered (Figure 1).

**Figure 2:**
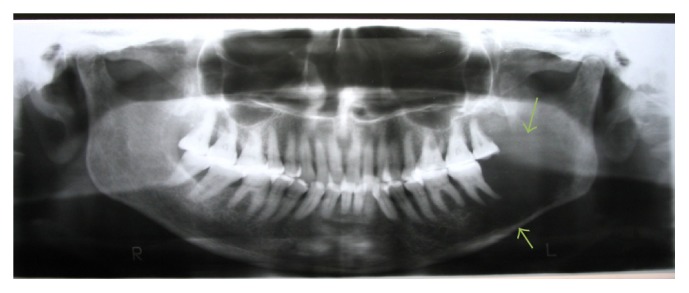
Preoperative orthopantomogram (arrow) showing diffuse irregular multilocular radiolucency in the left side of the lower jaw extending from parasymphysis region to the ramus of the mandible.

**Figure 3:**
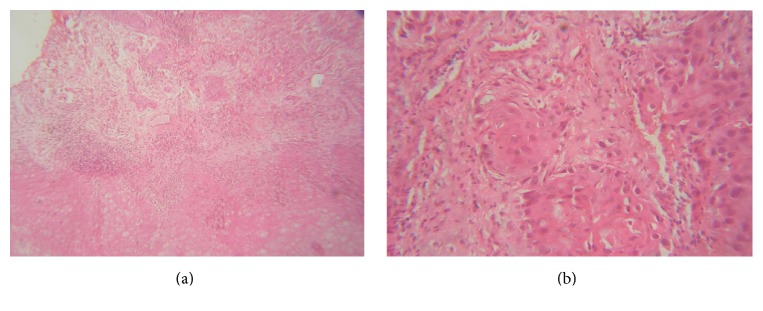
Microscopic image of the lesion exhibiting islands of tumor calls invading the underlying connective tissue stroma (H & E stain at 10x magnification; (a)) with features of nuclear and cytoplasmic atypia (H & E stain at 40x magnification; (b)).

Fine needle aspiration cytology (FNAC) revealed scanty fluid exhibiting sheets of cells with atypical features of hyperchromatic nucleus, pleomorphism, and altered nuclear cytoplasmic ratio suggestive of malignancy. Orthopantomogram (OPG) (Figure 2) view showed an extensive multilocular radiolucency in the left mandibular body extending from 33,34 region posteriorly to the ramus of mandible suggestive of malignancy. Chest radiograph appeared normal. Incisional biopsy revealed proliferative stratified squamous epithelium with dysplastic squamous islands exhibiting features of hyperchromatism, pleomorphism, and individual cell keratinization. The tumor cells showed no contact with the normal appearing overlying mucosa (Figures 3(a) and 3(b)).

Based on all findings, a final diagnosis of Primary Intraosseous Carcinoma of the mandible was given. Patient was treated by routine surgical removal of the lesion followed by postoperative radiotherapy (routine radiotherapy; 60 Grays) to prevent the likelihood of metastasis and poor outcome. Patient is under periodic follow-up till date without any evidence of malignancy.

## 3. Discussion

Jaw bones are the most common sites for odontogenic cyst and tumors affecting human skeleton. Primary Intraosseous carcinoma (PIOC) is most aggressive type of squamous cell carcinoma affecting the jaw. It is most aggressive type of squamous cell carcinoma affecting the jaw. Revised World Health Organization (WHO-1992)* (Waldrum and Mustoe) *classification has categorized PIOC broadly into the following types [6].

Type 1: PIOC ex odontogenic cyst
Type 2a: malignant ameloblastoma
Type 2b: ameloblastic carcinoma arising de novo ex ameloblastoma or ex odontogenic cyst
Type 3: PIOC arising de novo
  (i) Keratinizing
  (ii) Nonkeratinizing
Type 4: intraosseous mucoepidermoid carcinoma

Present tumor was found to arise from a surgical extracted site of the jaw. Therefore, the absence of initial connection with the ulcer in the overlying mucosal epithelium and distant primary tumor by physical or radiographic examination concludes the present case to be a squamous cell carcinoma arising de novo from the mandible.

PIOC arises from the epithelial remnants of odontogenesis, retained tooth germ, reduced enamel epithelium, Hertwig's epithelial root sheath, and epithelial remnants. These epithelial remnants proliferate in the presence of unknown stimuli into squamous cell carcinoma (SCC). A unique feature of this type of SCC is that they are infrequently associated with habits of alcohol, betel quid, and tobacco usage along with inflammatory stimulus with/without genetic factor being major risk factor [7].

PIOC occurs in 6th-7th decades of life and with an increased male predominance (3 : 1) [8] and mandibular posterior and maxillary anterior region being predominant site [9]. This case is of 66-year-old female patient with the lesion located in the mandible partially consistent with the previous findings. PIOC presents clinically as silent tumors to large lesions causing pain, jaw fractures, sensory nerve abnormalities, tooth mobility, trismus, intact mucosal surface, and lymphadenopathy [10]. This case can be renamed as “silent killer” due to the absence of marked clinical features except trismus, paresthesia, and intact mucosal surface.

In radiographic examination, PIOC appear as extensive unilocular to multilocular radiolucent lesions with periphery raggedness. Destruction of the vital structures within the jaw bones along with “floating teeth” appearance is seen. The surrounding cortical plates remain intact in addition to the abovementioned routine features making this reported case of PIOC unique.

Histopathological sections of this lesion reveal nests and islands of tumor cells with features of atypical nuclei, pleomorphism, dyskeratosis, and mitotic figures. There was no contact with the above mucosal epithelium. Focal areas of necrotic bone and varying degree of lymphoplasmacytic infiltration are evident. In this case, carcinoma showed an intimate connection to the surgical extracted site. There was no preexisting odontogenic cyst in the patient. Furthermore, clinically and histologically, no evidence of direct contact with oral mucosa is seen.

Modalities of treatment in PIOC may vary from wide resective surgery, radiotherapy or chemotherapy depending on adjuvant factors taken into consideration [4]. In the present case, this aggressive and rapidly expanding lesion was referred further for oncological opinion and management. These lesions should be planned well for treatment as they present with a poor prognosis with 30%–40% 5-year survival rate only [2].

Differential diagnosis of PIOC includes several odontogenic cysts and malignant odontogenic tumors, squamous cell carcinoma of mucosal origin, and metastatic lesions. To distinguish PIOC from the abovementioned neoplasms, diagnostic criteria include intact oral mucosa with absence of surface ulceration, tumors in physical and radiographic examination at the time of diagnosis, and six-month survival with no evidence of occult primary or negative autopsy.

The present case describes extra oral swelling with paresthesia, trismus, and intact mucosal surface. Radiology and histopathology revealed squamous cell carcinoma exclusively arising from mandible. Exclusion of any primary or metastatic deposits was fulfilled too. Thus, this case appears to be unique with a puzzling change to a dental surgeon.

We presented a unique case of PIOC of the mandible exhibiting as a silent invader with marked destruction. Even though the diagnosis of PIOC was rare, we insist that this lesion must always be included in the differential diagnosis of radiolucent lesions. This lesion must be biopsied immediately and patient must be closely observed during follow-up. We assume this article adds on knowledge and better understating to the target readers regarding various phases of this lesion.
